# Attenuated Microcirculation in Small Metastatic Tumors in Murine Liver

**DOI:** 10.3390/pharmaceutics13050703

**Published:** 2021-05-12

**Authors:** Arturas Ziemys, Vladimir Simic, Miljan Milosevic, Milos Kojic, Yan Ting Liu, Kenji Yokoi

**Affiliations:** 1 Houston Methodist Research Institute, Houston, TX 77030, USA; mkojic@houstonmethodist.org (M.K.); yliu4@houstonmethodist.org (Y.T.L.); kyokoi@houstonmethodist.org (K.Y.); 2 Bioengineering Research and Development Center BioIRC Kragujevac, 3400 Kragujevac, Serbia; vsimic@kg.ac.rs (V.S.); miljan.m@kg.ac.rs (M.M.)

**Keywords:** metastases, liver lobule, perfusion, diffusion, therapeutic resistance

## Abstract

Metastatic cancer disease is the major cause of death in cancer patients. Because those small secondary tumors are clinically hardly detectable in their early stages, little is known about drug biodistribution and permeation into those metastatic tumors potentially contributing to insufficient clinical success against metastatic disease. Our recent studies indicated that breast cancer liver metastases may have compromised perfusion of intratumoral capillaries hindering the delivery of therapeutics for yet unknown reasons. To understand the microcirculation of small liver metastases, we have utilized computational simulations to study perfusion and oxygen concentration fields in and around the metastases smaller than 700 µm in size at the locations of portal vessels, central vein, and liver lobule acinus. Despite tumor vascularization, the results show that blood flow in those tumors can be substantially reduced indicating the presence of inadequate blood pressure gradients across tumors. A low blood pressure may contribute to the collapsed intratumoral capillary lumen limiting tumor perfusion that phenomenologically corroborates with our previously published in vivo studies. Tumors that are smaller than the liver lobule size and originating at different lobule locations may possess a different microcirculation environment and tumor perfusion. The acinus and portal vessel locations in the lobule were found to be the most beneficial to tumor growth based on tumor access to blood flow and intratumoral oxygen. These findings suggest that microcirculation states of small metastatic tumors can potentially contribute to physiological barriers preventing efficient delivery of therapeutic substances into small tumors.

## 1. Introduction

Despite advancements in anticancer treatments, advent of immunotherapies, and improved diagnostics, metastatic disease remains the leading cause for 90% of cancer-related deaths [1]. The low therapeutic efficacy of anti-cancer treatments for metastatic tumors negatively impacts patient survival. While many types of primary tumors can be imaged and resected due to their size, it is challenging to even detect the metastases spreading from the primary site to distant organs which are made of individual circulating cancer cells (CTCs) or small CTC clusters [2,3]. The majority of CTCs are eliminated by the immune system of the host, but some can circulate in the bloodstream to reach and reside in the distant organs eventually becoming secondary tumors. Detection of such small cells is beyond current imaging capabilities due to a limited resolution of the modern clinical non-invasive diagnostic tools [4]. Thus these cells or cell clusters at distant organs, frequently referred to as micrometastases in clinics, can escape their detection [5].

These tiny secondary tumors may only be treated therapeutically on a systemic basis while also acknowledging common resistance mechanisms [6]. Drug biodistribution by the cardio-vascular system and drug extravasation/permeation into tissue microenvironment are key factors in delivering chemotherapeutic or immunotherapeutic drugs to cancer cells inside tumors. Nanoformulations were shown to be effective in exploiting Enhanced Retention and Permutation (EPR) effect in murine models. However, this effect is not widely present in humans and such nanoformulated drugs do not always reach their desired potential [7,8]. These formulations exploit the features of the capillary bed found in primary tumors; however, the small metastatic tumors may have no vasculature at times and different drug delivery strategies should be considered.

Inadequate understanding of microcirculation in metastases could be one of the reasons that treatments of metastatic disease fail. Limited knowledge of drug delivery coupled with scarce literature on the circulation in micrometastases contributes to poor outcomes in surviving metastasis disease. It is likely that there is a common assumption that drugs should reach those tiny tumors without obstacles and drug delivery challenges are impulsively dismissed in research questions. However, some of our studies conducted on murine breast cancer liver metastases have undoubtedly indicated that drug permeation into metastatic tumors is fundamentally hindered [9,10]. The metastatic tumors ranging from 50 to >600 µm in size did not show doxorubicin inside the tumors; instead, most of doxorubicin was presented in liver tissue surrounding those tumors. Drug permeation was not observed at any point of time for up to several days and the treatment did not affect tumor progression: Nontreated and treated animal groups have shown the same tumor progression. The common determinant of this therapeutic outcome is the absence of drug in tumors. Even though our immunohistochemical imaging and intravital microscopy indicates the presence of vasculature, the tumor capillaries were not functioning due to compromised blood flow [9].

Some studies have indicated that establishment of micrometastases may alter hepatic perfusion (e.g., the presence of ~500 µm size metastases in rats decreased hepatic perfusion on the level of an organ by up to 25%) [11]. Other studies have also found that the presence of metastases could significantly reduce liver perfusion in rats [12]. However, these results cannot explain the perfusion patterns present in and around metastases. One study investigated the intratumoral capillary perfusion in colon cancer liver metastases as a function of tumor progression [13]. Those results indicated that small tumors start as avascular but developed vascularization and perfusion during their progression above 500 µm in tumor size. Our previous study on breast cancer liver metastases [10] did not reveal evidences of perfusion within these tumor dimensions, but the differences in tumor models should be noted. Other study using casting techniques indicated that small metastases in the liver start in the liver lobules and eventually develop arterial supply only when tumors grew above 800–1000 µm [14]. While the overall number of studies is small, we one can notice that only larger tumors develop perfusion. However, the critical size at which tumors start angiogenesis and getting perfused can be different in different animal and tumor models. Thus, the question of how drugs reach those small tumors becomes even more relevant.

This study extends our focus on drug delivery challenges to liver metastases by conducting simulations of blood perfusion and oxygen permeation to metastatic tumors. The computational models inspired by our previous published in vivo results seek to understand better what physiological factors could be important in this problem. Our main goal is to understand what local microcirculation patterns are developed at different tumor locations in the liver lobule and how these patterns may influence drug delivery. 

## 2. Material and Methods

### 2.1. Computational Perfusion and Diffusion Analysis

Perfusion and diffusion were simulated by using our developed Composite Smeared Finite Element (CSFE) method [15,16], which employs the concept of smeared physical fields, like fluid flow, molecule transport by convection and diffusion through capillary system and tissue, or capillary density. Fluid flow within capillaries is governed by the Hagen–Poiseuille law, while in tissue it is modeled according to the Darcy pressure–velocity relationship. It is assumed that the Fickian law is applicable for diffusion. The 1D transport in capillaries is transformed into a continuum format. The material parameters of the model include fluid viscosity, hydraulic permeability of the capillary walls, Darcy coefficient for fluid flow in tissue, and diffusivity in capillaries and tissue for diffusion.

**Figure 1:**
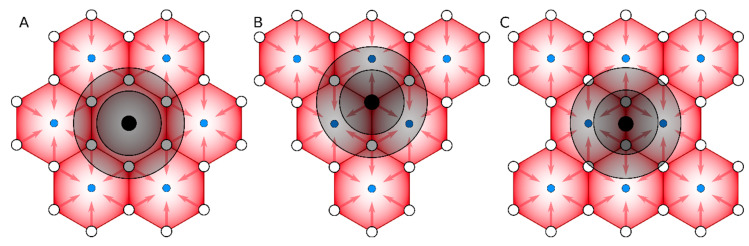
Schematics of three representative models of small metastatic tumors in the liver at different locations with respect to liver lobule structure: (**A**) central vein, (**B**) portal vein, and (**C**) acinus. Portal vessels are represented by open circles and portal veins by blue filled circles. Red gradient illustrates the O_2_ gradients and red arrows represent blood flow direction from portal vessel toward central veins. The black circles represent tumors with different radii (50, 200, 350 µm) to illustrate how tumor size interferes with local blood microcirculation during tumor progression.

Finite Element (FE)-based and other Computational Fluid Dynamics (CFD) methods were already successfully applied to study hepatic circulation, including in a human liver [17,18,19]. The SCFE method simulated local perfusion and diffusion fields in the vicinity of a small tumor embedded in different locations with respect to liver lobule with lobule side distance of 250 µm (representative distance between portal vessels in liver lobule hexagon). Each model has common key components: sinusoidal capillaries, liver tissue, portal vessels, and central vein. Blood flow was pressure-driven from portal vessels into the central vein by passing through capillaries of the sinusoidal space (Figure 1). The model was executed in 2D setup with tissue thickness set to 1 µm. 

Portal and central vessels were modeled explicitly with assigned vessel diameters of 40 and 25 µm, and local blood pressures of 250 and 0 Pa, that is a driving force of local hemodynamics in liver lobule. The portal vessel triad was simplified into one main vessel. The pressure difference was adjusted to representative values found in in vivo studies [20]. The sinusoidal lobule domain was simulated with the smeared vascular and tissue domains. The sinusoidal capillary domain was simulated with the volumetric capillary fraction r_V_ = 0.4, the capillary diameter of 8 µm, and the capillary wall thickness of 1 µm. Blood viscosity was set to 0.001 Pa·s. The hydraulic permeability of capillary walls was set to zero because the pressure gradient in sinusoidal space is only present along capillary length and our previous studies indicated clearly that were effectively no perfusion inside small metastatic tumors in liver [9,10].

We have built three representative perfusion models, where small metastatic tumors get initiated in the central vein (Figure 1A), portal vein (Figure 1B), and acinus (Figure 1C). If the tumor domain overlapped with portal or central vessels in liver lobules, those vessels were eliminated from the model. Capillaries inside tumors were modeled in the same way as in the liver domain, just different r_V_ values were assigned as it is explained further. Blood viscosity in tumors was set to 0.003 Pa·s to mimic vascular tortuosity effects.

Oxygen (O_2_) diffusion was simulated to understand the impact of the changes of local microcirculation upon the presence of tumors. The diffusion coefficient of O_2_ was set to 1000 µm^2^/s, which is 2.5 times lower from its diffusivity in water to reflect potential effects from dense tissues [21,22]. Arterial O_2_ concentration is 0.13 mM [23] or 0.13 amol/µm^3^; however, because it is mixed approximately at a 1:4 ratio with venous blood in the portal structure, O_2_ concentration in portal vessels was set to 0.085 amol/µm^3^. O_2_ consumption by tissues was simulated by introducing a sink term in tissue domains with representative consumption rates of 0.03 in tumors and 0.05 in liver [23,24].

### 2.2. In Vivo and Imaging Experiments

The in vivo and imaging data that were used in this study for the comparison to simulations were published in [10]. Here we provide only a brief summary of those experiments for the benefit of readers. Experimental liver metastases were created by injecting 4T1 breast cancer cells (1 × 105/100 µL) into the spleen bodies of female Balb/C mice (6–8 weeks of age). Later, mice were intravenously injected with 6 mg/kg PLD (Doxoves-Liposomal Doxorubicin HCl; FormuMax Scientific Inc., Sunnyvale, CA, USA) and sacrificed 24 h later. The liver was excised and processed to image endothelial cells, nuclei, and PLD accumulation. The surgical procedures have been approved by the Institutional Animal Care and Use Committee (IACUC) of Houston Methodist Research Institute. Immunofluorescent staining of endothelial cells and nuclear staining were performed using antibodies to CD31 and DAPI, respectively. The fluorescence of doxorubicin was imaged at the excitation and emission wavelengths of 488 and 590 nm. The images were captured using a confocal laser-scanning microscope.

## 3. Results

We simulated blood perfusion and O_2_ diffusion patterns in and around small metastatic tumors in the liver seeking to understand how the establishment of a tumor in specific liver lobule locations can affects the perfusion of tumors, the microenvironment, and O_2_ concentration. As the liver lobules comprise characteristic locations such as the portal vein, central vein, and acinus, we simulated tumors of different sizes at those locations with different vascular fractions to mimic avascular and vascular states of tumors. Different r_V_ values were investigated as a result of our previous findings, which elucidated that tumor capillarization can be heterogeneous despite the presence of nonperfused capillaries in the tumors [9,10]. Thus, diffusion may become the key determinant for the fundamental mass exchange mechanisms at the microenvironment level. The simulation of avascular tumors (r_V_ = 0) in an idealized symmetric vascular configuration and vascularized tumors (r_V_ = 0.05), in both an idealized symmetric avascular and vascular configuration, were performed by imposing 20% randomized shift in x- and y-coordinates of portal and central vessels. To model tumor progression effect on perfusion and diffusion patterns, we simulated tumors of different radii (R) in the range 50–350 µm.

**Figure 2:**
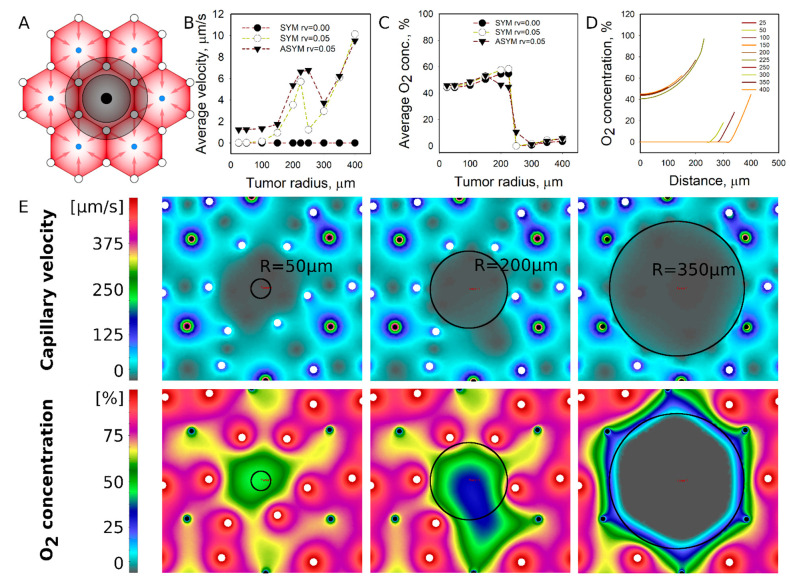
Simulated perfusion and diffusion patterns for the metastatic tumor established at the location of the central vein. (**A**) The general schematic of tumor location (see Figure 1). (**B**) Average tumor perfusion velocity as a function of the tumor size for different tumor cases: avascular tumors with idealized vessel configurations, vascularized (r_V_ = 0.05) tumors in idealized vascular configuration, and vascularized tumors in non-idealized (asymmetric) vascular configuration. (**C**) Average O_2_ concentration as a function of tumor size. (**D**) O_2_ concentration profiles inside the tumor. (**E**) Flow velocity and O_2_ concentration fields at specific tumor sizes with asymmetric portal and central vein locations (open circle—portal vessels, dark circles—central veins).

Central vein location: Tumors established at the central vein location obstruct the drainage of blood from the six surrounding portal vessels and further immobilize the function of the central vein (Figure 2A). This situation implies that the blood flow is compromised inside the lobule where the source of O_2_ and nutrients delivery to the tumors are solely dependent on diffusion mechanisms. The perfusion of a tumor was estimated by calculating the mean flow velocity in the tumor domain; results are plotted in Figure 2B. While blood flow velocities in the liver were at 50–100 µm/s, blood flow velocities in tumors were effectively absent for small tumors with R < 150 µm. Only when the tumor radius approached 200 µm and the tumor became comparable in size with the liver lobule, the average tumor perfusion velocity increased to 6 µm/s. The perfusion of larger tumors that engulfed the nearest portal vessels is lost. The average O_2_ concentration profile in tumors was different to the flow velocity profile. Tumors with R = 50–200 µm had about ~50% of the O_2_ concentration that was provided by portal vessels, but the O_2_ concentration substantially dropped when the tumor exceeded the lobule size (Figure 2C). The O_2_ concentration profiles in the tumors are plotted in Figure 2D, showing that O_2_ concentration is almost same for tumors with smaller than the lobule. However, once the nearest portal vessels are eliminated as the tumor grows, hypoxic conditions are generated due to the lack of nutrient supply from its surrounding non-functional portal veins. The flow and O_2_ concentration fields are shown in Figure 2E to show a poorly perfused tumor with low O_2_ concertation fields surrounding or inside the tumor during its progression. Interestingly, a non-ideal position of portal and central vessels contributes to better perfusion patterns when compared to ab idealized lobule structure. However, there is little effect on the O_2_ field inside the tumor.

**Figure 3:**
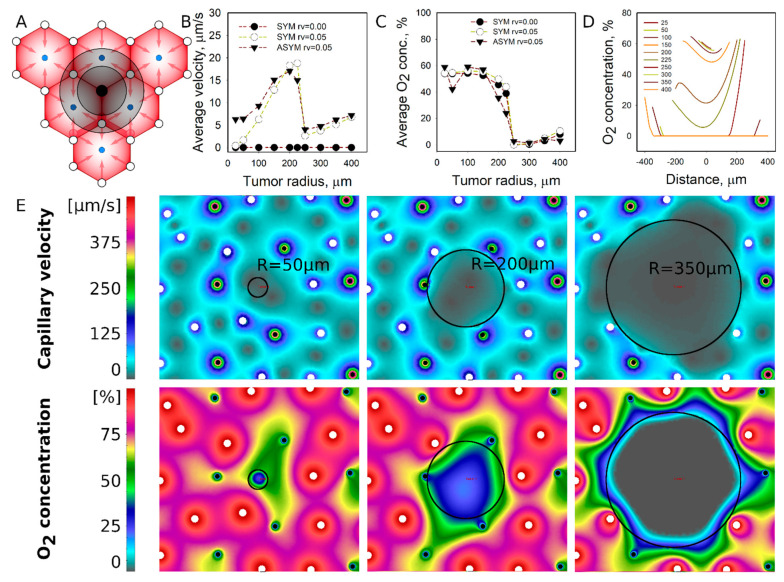
Simulated perfusion and diffusion patterns for the metastatic tumor established at the location of portal vessels. (**A**) The general schematic of tumor location (see Figure 1). (**B**) Average tumor perfusion velocity as a function of the tumor size for different tumor cases: avascular tumors with idealized vessel configurations, vascularized (r_V_ = 0.05) tumors in idealized vascular configuration, and vascularized tumors in non-idealized (asymmetric) vascular configuration. (**C**) Average O_2_ concentration as a function of tumor size. (**D**) O_2_ concentration profiles inside the tumor. (**E**) Flow velocity and O_2_ concentration fields at specific tumor sizes with asymmetric portal and central vein locations (open circle—portal vessels, dark circles—central veins).

Portal vein location: Tumors established at the portal vessel location hinder the function of the portal vein and obstruct the blood supply of the vessels (Figure 3A). In contrary to the location of the central vein, the tumors surrounded by portal and central vessels are able to maintain the perfusion and nutrient supply outside the tumor. The calculated flow velocities in tumors are close to zero for tumors with R = 50 µm but increases to 15–20 µm/s for tumors approaching the size of the liver lobule. Similar to the tumors in the central vein location, flow drops once tumor size gets larger than the lobule (R > 250 µm) and further eliminates its surrounding lobule vessels. The O_2_ concentration is present inside the tumors with R < 250 µm; however, it drops to zero for larger tumors (Figure 3C). The O_2_ concentration profiles show that the O_2_ level gradually changes inside a tumor as its hypoxic condition arises; especially when the size exceeds the lobule radius. Perfusion and centration fields (Figure 3E) reveal deficiencies in flow velocities and O_2_ concentration compared to the tumor surroundings.

**Figure 4:**
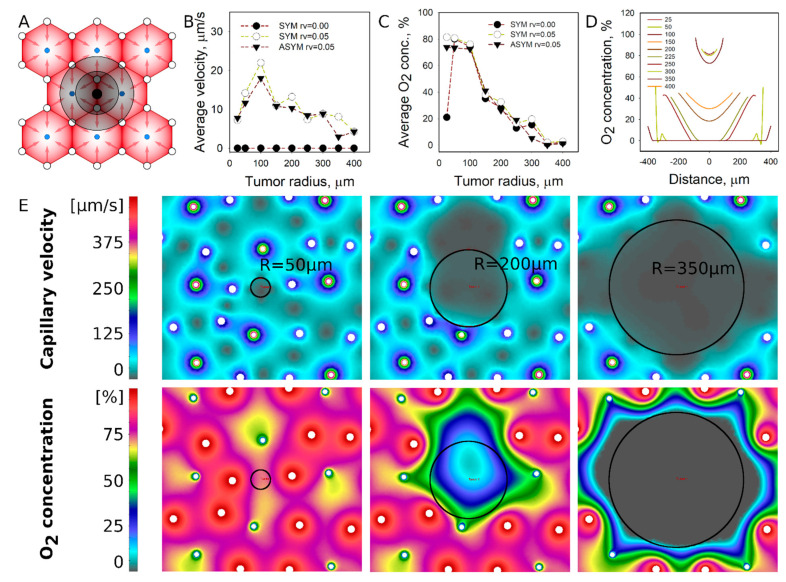
Simulated perfusion and diffusion patterns for the metastatic tumor established at the acinus location. (**A**) The general schematic of tumor location (see Figure 1). (**B**) Average tumor perfusion velocity as a function of the tumor size for different tumor cases: avascular tumors with idealized vessel configurations, vascularized (r_V_ = 0.05) tumors in idealized vascular configuration, and vascularized tumors in non-idealized (asymmetric) vascular configuration. (**C**) Average O_2_ concentration as a function of tumor size. (**D**) O_2_ concentration profiles inside the tumor. (**E**) Flow velocity and O_2_ concentration fields at specific tumor sizes with asymmetric portal and central vein locations (open circle—portal vessels, dark circles—central veins).

Acinus location: This location is different from portal or central vessels because it does not occlude the lobule vessels, as the smaller tumor sizes did not change the perfusion patterns surrounding the tumor (Figure 4A). This location is also characterized by a lower flow velocity in the liver lobule. The largest tumor perfusion of 20 µm/s was calculated for the tumor with a radius of 100 µm (Figure 4B). However, flow velocities of 100 µm/s were calculated for the most of the tumors. The heterogeneity of spatial positions of lobule vessels did not affect perfusion as significantly as in the locations of central or portal vessels.

Tumors with R < 100 µm were well oxygenated and showed monotonic reduction of O_2_ concentration with an increase in size (Figure 4C). Substantial hypoxia was developed for the tumors with R > 200 µm (Figure 4D). The flow velocity and O_2_ concentration fields that are depicted in Figure 4E showing that small tumors are embedded well in O_2_ concentration field; however, larger tumors with R > 250 µm develop O_2_-deprived tissues inside and around the tumor.

**Figure 5:**
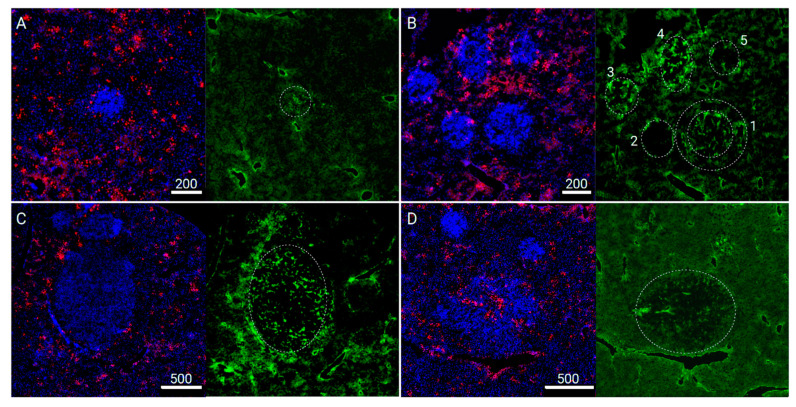
Chemotherapeutic drug doxorubicin extravasation patterns in murine liver tissues with 4T1 breast cancer metastases (blue—cell nuclei stained with DAPI, red—doxorubicin, green—vasculature stained in CD31. Characteristic drug extravasation patterns could be correlated to the perfusion patterns around tumors established at different locations of lobule (see text). (**A**)–tumors could be similar to the tumors established at the acinus or portal vessel locations (scale = 200 μm); (**B**)–tumors originating in the area of the central vein (scale = 200 μm); (**C**)–large metastatic tumor without drug extravasation (scale = 500 μm); (**D**)–drug perfusion and extravasation observed in large tumors only if they had large vessels developed (scale = 500 μm).

Comparison of simulated perfusion and drug extravasation patterns. Figure 5 shows some tumor and fluorescent therapeutic extravasation patterns in murine liver from our previous study, where experimental breast cancer liver metastasis were employed by using the 4T1 cell line in the Balb/c murine animal model [10]. The study investigated the pegylated liposomal doxorubicin (PLD) biodistribution in the liver and in small metastatic tumors during cancer progression. In-depth quantitative immunofluorescent imaging analysis found high heterogeneity of transport properties of tumors, including tumor vascularization and a lack of doxorubicin inside tumors. The absence of drug in tumor was observed despite the presence of intratumoral capillaries. Our other study using intravital imaging found no perfusion in liver metastases, although capillaries were present based on immunofluorescent imaging [9]. Both studies led to the question about the origins of failing tumor perfusion. Red fluorescent drug doxorubicin (Figure 5) can serve as a surrogate marker to be compared with simulated patterns of perfusion and O_2_ centration (Figure 2, Figure 3 and Figure 4). While this correlation is circumstantial, the comparison establishes contextual relation allowing to judge if there are commonalities between what we simulate and what we observe. 

Figure 5A shows a small tumor with doxorubicin fluorescence present around the tumor but not inside, which could be similar to the tumors established at the acinus or portal vessel locations. Figure 5B shows many tumors in proximity, which makes it difficult to differentiate the tumor type based on the location. However, there is a tumor (#1 in the Figure 5B) that we could identify the peripheral ring with the absence of doxorubicin fluorescence, which can be classified as the tumors originating in the area of the central vein (Figure 2). Our visual inspection of immunohistochemical images could identify only few instances of such tumors, which further confirmed our assumptions for the less CTC-arresting central vein location when compared to other investigated locations. Figure 5C shows a large metastatic tumor, which could be expected to be vascularized and perfused because of its size; however, it does not show any drug inside the tumor. Similarly, the tumors displayed in Figure 5A,B have endothelial cells present in all of them. The few instances we could observe drug perfusion and extravasation happened in large tumors only if they had large vessels developed (Figure 5D).

## 4. Discussion

The results of our study suggested that microcirculation patterns in tumors and their surrounding microenvironment can be drastically altered even by the smallest tumors. We have explored three representative locations of metastases. The locations of portal and central vessels correspond to the boundary region between larger vessels and sinusoid capillaries, where vessel lumen changes from larger to small (portal vessels) and from small to larger (central vein). The acinus location is different due to its position in between the two portal and central vessels where local pressure gradients and flow velocities are lower with a possibility of CTC accumulation. Comparing all three locations, we expect the tendency of arrested CTC would diminish in the following order: portal vessels, acinus, central vein.

The microcirculation patterns inside tumors have one similarity: Blood flow is reduced inside tumors compared to the surrounding in the liver. Whereas the liver is a very porous organ with a high volumetric capillary fraction, compared to tumors, leading to higher flow rate outside tumors. Furthermore, after tumors are established, their size is smaller than the liver lobule and these tiny tumors remain relatively far away from feeding vessels (e.g., portal vessels). At the same time, tumor establishment blocks the flow and creates a partially hypoxic environment inside and outside of the liver lobules. In the early phase of tumor growth, a small tumor does not depend on vessels for nutrient supply because diffusion can serve them well. It is possible that despite the developed hypoxia, which triggers angiogenesis, the interior of tumors may not have enough of a pressure gradient to provide efficient flow and mechanically stimulate vessel development. Even if vessels were developed by angiogenesis induced by hypoxia and biological signaling, the blood pressure inside capillary lumen of tumors may be insufficient to counteract the mechanical pressure inside tumor leading to collapse of capillary lumen which corroborates with other studies indicating that pressure is key in hepatic microcirculation [17]. We have already shown by using immunohistochemistry, intravital imaging, and fluorescent therapeutic perfusion that capillaries are present in small metastatic tumors, whereas blood flow is absent [9]. 

Heterogeneity of pure physical origin and its effect on biology is little studied, while biological heterogeneity is widely researched [25]. Our analysis shows an interesting manifestation of physical heterogeneity that could potentially alter the perfusion in metastatic tumor. We have investigated idealized configurations of portal and central vessel and configurations of vessels that were slightly randomized to mimic biological reality. Non-ideal or slightly asymmetric configurations led to slightly larger flow velocities inside tumors, most probably because of the development of larger local pressure gradients. However, it had no effect on the O_2_ concentration field inside and around the tumors. Furthermore, our analysis shows that the physical location can be a factor in determining the interaction between the tumors and its microenvironment. The microcirculation differences are due to distinct tumor locations with respect to the levels of functioning blood vessels which can result in different progression patterns; potentially instigating different biological cues, including therapeutic resistance.

Micrometastatic tumor circulation is still being investigated, while circulation in primary tumors has been under investigation for decades [26]; especially in the context of anti-angiogenic treatments [27]. Antivascular treatments prevent tumors from obtaining nutrient supply because primary tumors at the time of their detection are well established and most of them are well vascularized tissues. However, the metastatic tumors are found to be at least two to three orders of magnitude smaller than the primary tumor in size and can be avascular. Many anti-vascular strategies and theories were developed for primary tumors, like the well-known ‘normalization’ theory [28], which can be difficult to apply to the micrometastases due to their differences from the primary tumors. The key difference is the lack of circulation, at least in the current example of the 4T1 breast cancer metastases in this study. In order to facilitate the delivery of drugs to these small tumors, the development of vasculature could be a crucial condition and all anti-vascular strategies could make the treatment of early metastatic disease worse. 

## 5. Conclusions

The microcirculation simulation results have revealed that newly established small metastatic tumors in the liver may alter local microcirculation around them in the vicinity of the nearest liver lobules. Those changes also lead to poor perfusion of tumors. We have investigated a few anatomically distinct locations of tumors with respect to lobule main vessels and have found microphysiological differences among them in term of their perfusion and access to O_2_. Tumors established at acinus do not disrupt local microcirculation; therefore, they are well perfused with high O_2_ concentrations. Considering that the metastatic process potentially seeds many metastatic tumors, it is possible that local microcirculation patterns eventually transcend their heterogeneity into tumor biology eventually. These results confirmed our previous findings that tiny metastatic tumors are not perfused despite the presence of endothelial cells and vasculature, offering some clues that biophysics of microcirculation can be at least partially responsible for that. Our findings indicate that the local microenvironment of small metastases does not possess sufficient blood pressure gradients to perfuse tumors effectively. We speculate this may potentially lead to tumor capillary collapse as a result of insufficient pressure gradients in the local microcirculation to overcome tumor mechanical pressure. These findings have important implications in the treatment of metastatic disease, as it demonstrates that poor microcirculation in and around micron-size metastases may cause one more physiological barrier to the delivery of therapeutics, which is widely unacknowledged. While this study was performed on murine liver, future analysis on human tissues are needed in order to determine the physiological barriers and parameters in metastatic disease.

## Data Availability

Not applicable.
